# Dysregulation of N-terminal acetylation causes cardiac arrhythmia and cardiomyopathy

**DOI:** 10.21203/rs.3.rs-3398860/v1

**Published:** 2024-07-19

**Authors:** Vassilios bezzerides, Daisuke Yoshinaga, Rui Feng, Maksymilian Prondzynski, Kevin Shani, Yashasvi Tharani, Joshua Mayourian, Milosh Joseph, David Walker, Raul Bortolin, Chrystalle Carreon, Bridget Boss, Shelia Upton, Kevin Parker, William Pu

**Affiliations:** Boston Children’s Hospital; Boston Children’s Hospital; Boston Children’s Hospital; Boston Children’s Hospital; Harvard School of Engineering and Applied Sciences; Boston Children’s Hospital; Boston Children’s Hospital; Boston Children’s Hospital; Boston Children’s Hospital; Boston Children’s Hospital; Boston Children’s Hospital; Dartmouth Hitchcock Medical Center; Dartmouth Hitchcock Medical Center; Harvard University; Boston Children’s Hospital

## Abstract

N-terminal-acetyltransferases including NAA10 catalyze N-terminal acetylation (Nt-acetylation), an evolutionarily conserved co-translational modification. Little is known about the role of Nt-acetylation in cardiac homeostasis. To gain insights, we studied a novel NAA10 variant (p.R4S) segregating with QT-prolongation, cardiomyopathy and developmental delay in a large kindred. Here we show that the NAA10-R4S mutation reduced enzymatic activity, decreased expression levels of NAA10/NAA15 proteins, and destabilized the enzymatic complex NatA. In NAA10R4S/Y-iPSC-CMs, dysregulation of the late sodium and slow rectifying potassium currents caused severe repolarization abnormalities, consistent with clinical QT prolongation. Engineered heart tissues generated from NAA10R4S/Y-iPSC-CMs had significantly decreased contractile force and sarcomeric disorganization, consistent with the pedigree’s cardiomyopathic phenotype. We identified small molecule and genetic therapies that normalized the phenotype of NAA10R4S/Y-iPSC-CMs. Our study defines novel roles of Nt-acetylation in cardiac regulation and delineates mechanisms underlying QT prolongation, arrhythmia, and cardiomyopathy caused by NAA10 dysfunction.

## Introduction

N-terminal-acetyltransferases (NATs) catalyze protein N-terminal acetylation (Nt-acetylation), an evolutionarily conserved co-translational modification that regulates protein degradation, protein-protein interactions, membrane targeting, and protein folding^1^. After the initiation of translation, methionine aminopeptidases excise the initiator methionine from 80% of mammalian proteins, creating a substrate for NATs to irreversibly transfer an acetyl group from acetyl-CoA. N-terminal-acetyltransferase A (NatA), one of five mammalian NATs with distinct substrate specificities^2^, preferentially acetylates G, S, A, T, or V residues exposed at protein N-termini by α-methionine removal^3^. By sequence analysis, NatA can modify up to 40% of expressed mammalian proteins, although there are few functionally validated targets^4^. Despite the diversity of potentially affected signaling pathways, little is known about the effects of Nt-acetylation in the heart.

NatA contains a catalytic subunit, NAA10, and a regulatory subunit, NAA15. Pathological variants in NAA10 cause NAA10-related syndrome, a rare multi-system disorder characterized by developmental delay, hypotonia, QT prolongation, arrhythmias, and increased mortality often before 1 year of age^5^. In addition to QT prolongation and sudden death, NAA10 variants are associated with hypertrophic cardiomyopathy and congenital heart defects including atrial septal defects, ventricular septal defects, and tetralogy of Fallot^5–9^. Located on the X-chromosome, pathogenic NAA10 variants are inherited in an X-linked recessive pattern, with severe clinical phenotypes manifesting predominantly in males^10,11^. Female carriers present with more variable manifestations that range from mild to severe developmental delay with or without cardiac involvement^8,10,12^. Patients with cardiac involvement more commonly have NAA10 variants within the N-terminally located NAA15 interaction or catalytic domains, whereas those with neurodevelopmental delays generally have variants located closer to the C-terminus^13^, suggesting the importance of NatA complex formation for cardiac homeostasis.

Recent large-scale exome sequencing projects of patients with congenital heart disease identified several *de novo* NAA15 variants^14^. Cardiomyocytes derived from human induced pluripotent stem cells (iPSC-CMs) with a single missense mutation (NAA15^R276W/WT^) or NAA15 haploinsufficiency (NAA15^WT/−^) demonstrated minor contractile defects under loaded conditions, whereas iPSCs lacking NAA15 had poor viability and did not readily differentiate into iPSC-CMs^14^. These data support the importance of NatA function in a broad range of cardiovascular diseases, but the underlying mechanisms and cardiac targets remained undefined.

Despite NAA10-related syndrome pointing to a key role of NAA10 and Nt-acetylation in human cardiac homeostasis, little is known about the underlying disease mechanisms. Here we addressed this question by using iPSC disease-modeling, bioengineering, cellular electrophysiology, and optogenetics. We identify the first cardiac-specific targets dysregulated by NAA10 dysfunction and provide novel mechanistic insights into the roles of Nt-acetylation in cardiac homeostasis and disease.

## Results

### Identification of novel NAA10 variant

**Figure 1:**
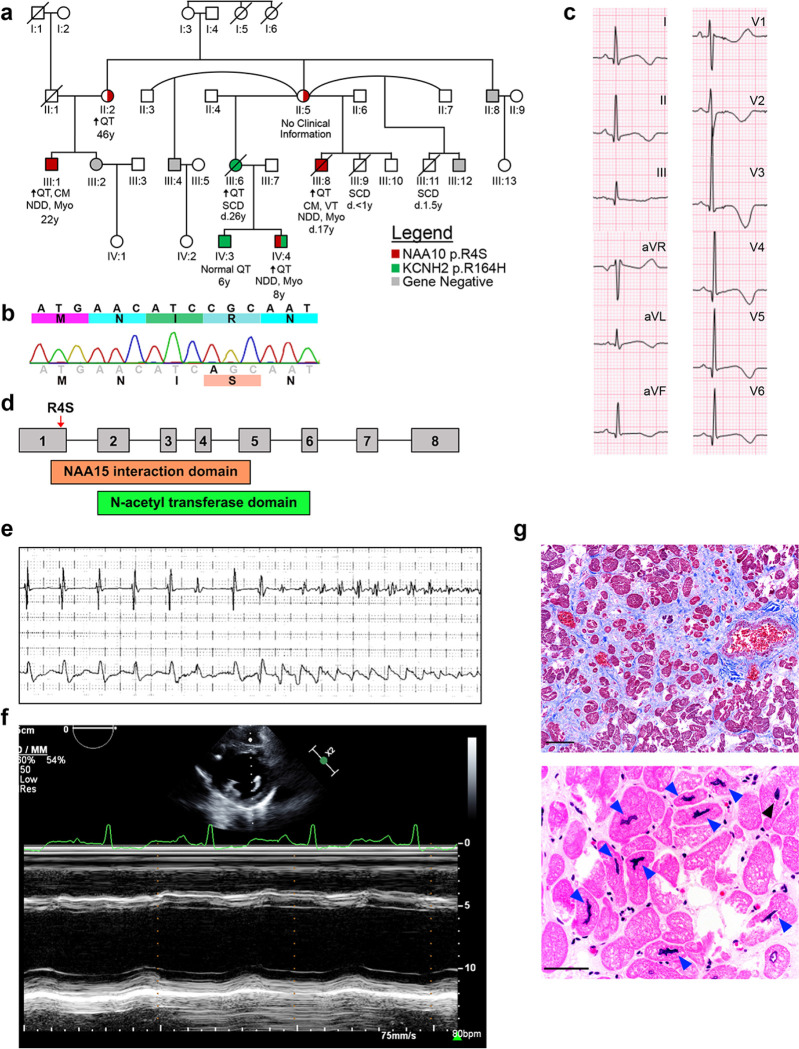
A novel NAA10 variant is associated with clinical long QT syndrome and severe cardiomyopathy. **a.** A four generation family pedigree identified predominantly male patients with QT prolongation, developmental delay, and early mortality in a family referred for “gene-negative long QT syndrome”. Clinical sequencing revealed the NAA10 p.R4S variant (red) to segregate with the clinical phenotype, whereas the variant of unknown significance in KCNH2 (p.R164H, shown in green) did not segregate with QT prolongation. In generation III: both male subjects (III:9, III:11) died as infants. Female (III:6) died at the age of 26 years from cardiac arrest prior to the availability of testing for NAA10. **b.** Sanger sequencing of the proband (indicated by arrow). **c.**ECG for patient III:8 with QT prolongation (QTc = 545 msec) and inverted T-waves. **d.** A schematic of the exon structure of the NAA10 gene overlaid with the novel p.R4S variant and its relationship to the domain structure of the NAA10 protein. **e.** Tracing from implantable cardiac defibrillator with short-long-short coupling prior to initiation of Torsades de Pointes (TdP). **f.** M-mode echocardiography of patient III:8. Calculated ejection fraction (EF) of 22.3%. **g.** Cardiac micrographs from patient III:8 post-mortem. Upper panel shows Masson Trichrome staining (scale bar = 100 μm) and lower panel is hematoxylin and eosin staining (scale bar = 50 μm) of the left ventricle. Arrows indicate abnormal appearing nuclei. Abbreviations for panel A: ↑QT = QTc prolongation, CM = Cardiomyopathy, d. = age of death, NDD = Neurodevelopmental delay, Myo = Peripheral skeletal myopathy, VT = Recurrent ventricular tachycardia, and SCD = Sudden cardiac death.

We evaluated a four-generation kindred with multiple members who had QT prolongation and sudden cardiac death (SCD) in young patients (Fig. 1a). Clinical genetic testing of the individuals with phenotypic QT prolongation for variants in congenital long QT syndrome (cLQTS) related genes revealed a variant of unknown significance in KCNH2 p.R164H (Clinvar, VCV000067508.8) that did not segregate with the disease phenotype (Fig. 1a). Expanded genetic testing for cardiomyopathies and skeletal myopathies in patients III:1 and III:8 also did not reveal any known variants. However in addition to QT prolongation and arrhythmia, male patients had significant neurodevelopmental delay and mild peripheral myopathy. These extra-cardiac phenotypes suggested a multi-system disorder. Extended genetic testing for variants associated with developmental delay identified a novel variant, *NAA10* c.10 C>A, p.R4S, that co-segregated with clinical manifestations in an X-linked recessive pattern in family members available for genetic testing (Fig. 1b). Male family members with the *NAA10*^*R4S/Y*^ variant had significant QT prolongation and T-wave abnormalities (Patient III:8, Fig. 1c, Extended Data Fig. 1). The NAA10^R4S/Y^ variant lies within the N-terminal NAA15 interaction domain necessary for formation of the NatA complex (Fig. 1d)^15^. Consistent with severe QT prolongation, patient III:8 developed frequent episodes of ventricular tachycardia despite high-dose beta-blocker therapy (nadolol 2.8 mg/kg/day), necessitating insertion of an implantable cardiac defibrillator (ICD) (Fig. 1e). All his episodes of ventricular arrhythmia occurred during rest or with only minimal physical activity. This patient developed progressive heart failure with an ejection fraction < 25%, LV dilation, and symptoms consistent with class IV NYHA heart failure^16^. Despite aggressive medical and supportive therapies, he died from complications of heart failure at 17 years of age (Fig. 1f). Histopathology of the post-mortem LV samples demonstrated prominent interstitial fibrosis, and hypertrophic cardiomyocytes with bizarre hyperchromatic nuclei and highly irregular nuclear contours (Fig. 1g). These clinical data support the identification of a novel NAA10 variant associated with severe QT prolongation, ventricular arrhythmias, and dilated cardiomyopathy.

### The R4S mutation impairs NatA catalytic activity.

**Figure 2:**
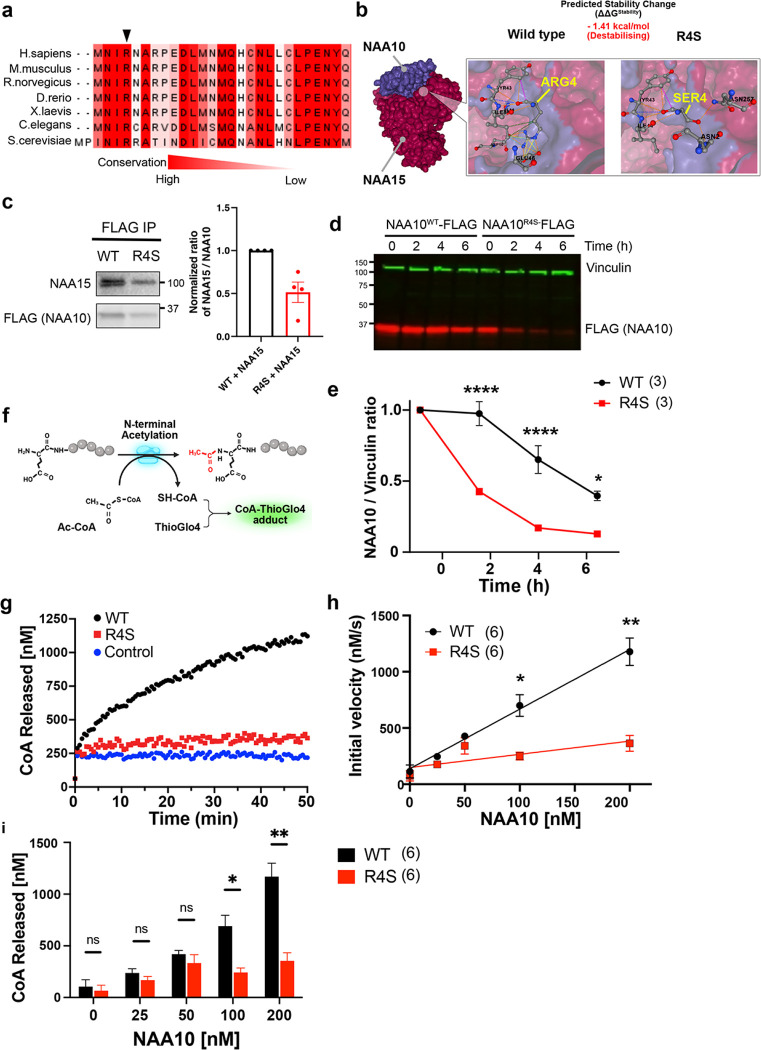
The R4S mutation induces NAA10 instability and decreases enzymatic function. **a.** Alignment of NAA10 protein sequences across multiple species. The arginine indicated by the arrow is highly evolutionarily conserved. **b.** HEK293 cells were co-transfected with NAA10-FLAG and NAA15 and lysates were immunoprecipitated with anti-FLAG antibody. Immunoprecipitants were separated by gel electrophoresis with subsequent immunoblotting using anti-NAA10 and anti-NAA15 antibodies. The binding capacity is quantified as a function of NAA10 protein levels (right panel). **c.** The R4S mutation alters NAA10 protein stability and possible interactions with NAA15 by *in silico* predication (DynaMut2). **d.** HEK293T cells transfected with NAA10^WT^-FLAG and NAA10^R4S^-FLAG were treated with cycloheximide for 0, 2, 4, and 6 hours and cell lysates were analyzed by western blotting with fluorescent secondary antibodies for vinculin and FLAG. **e.** Quantification of D, with normalization of NAA10/vinculin ratio to initial NAA10 protein levels. **f.** Schematic of ThioGlo4 assay to quantify the enzymatic activity of NAA10. Enzymatic production of CoA is detected by ThioGlo4 probe with excitation at 400 nm and emission at 465 nm. **g.** Representative traces of ThioGlo4 reaction over time indicating NAA10 activity. NAA10 WT or NAA10-R4S enzyme concentrations were 100nM. No enzyme was added to the control sample. **H.** The initial velocity of the enzymatic reaction was measured ThiolGlo4 fluorescence within the first 10 seconds. **I.** The total amount of CoA released was measured at 30 minutes after initiation of the enzymatic reaction. Statistics performed by one-ANOVA with Tukey’s post-hoc test for B. ****P <0.0001, **P<0.01, *P<0.05.

The arginine at position 4 (R4) is highly conserved, suggesting a key role in NAA10 function (Fig. 2a). *In silico* analysis of existing high-resolution structures of the NAA10-NAA15 complex^17^ predicted that the R4S variant would impair local hydrogen bonding and ionic interactions destabilizing NAA10 (Fig. 2b). To measure the effect of NAA10-R4S on NAA10-NAA15 interaction, we performed co-immunoprecipitation assays in transfected HEK293 cells. NAA10-R4S co-immunoprecipitated 50% less NAA15 than wild-type NAA10 (Fig. 2c). Furthermore, we noted that NAA10-R4S expressed less protein than wild-type (Extended Data Fig. 2a), leading us to suspect increased protein degradation. To assess protein half-life, we inhibited protein synthesis with cycloheximide and measured protein levels of expressed WT and mutant NAA10 proteins. NAA10-R4S displayed more rapid protein degradation than NAA10-WT when normalized to vinculin (Fig. 2d,e).

As the catalytic subunit of NatA, NAA10 catalyzes the transfer of acetyl-CoA to protein N-termini, releasing free CoA^12^. Free CoA reacts with ThioGlo4, forming a fluorescent adduct (Fig. 2f). We used this reaction to measure the effect of the R4S mutation on NAA10 catalytic activity. Monitoring ThioGlo4-CoA fluorescence over time showed that purified NAA10-R4S protein had significantly lower catalytic activity than NAA10-WT protein (Fig. 4g, h). Increasing protein concentration failed to overcome the enzymatic defect of NAA10-R4S even at the termination of the reaction (Fig. 2i). Collectively these data show that the novel mutation NAA10-R4S destabilizes the NatA complex, increases NAA10 protein degradation, and directly impairs NAA10 enzymatic activity.

### Prolonged repolarization in *NAA10*^*R4S/Y*^-iPSC-CMs

**Figure 3:**
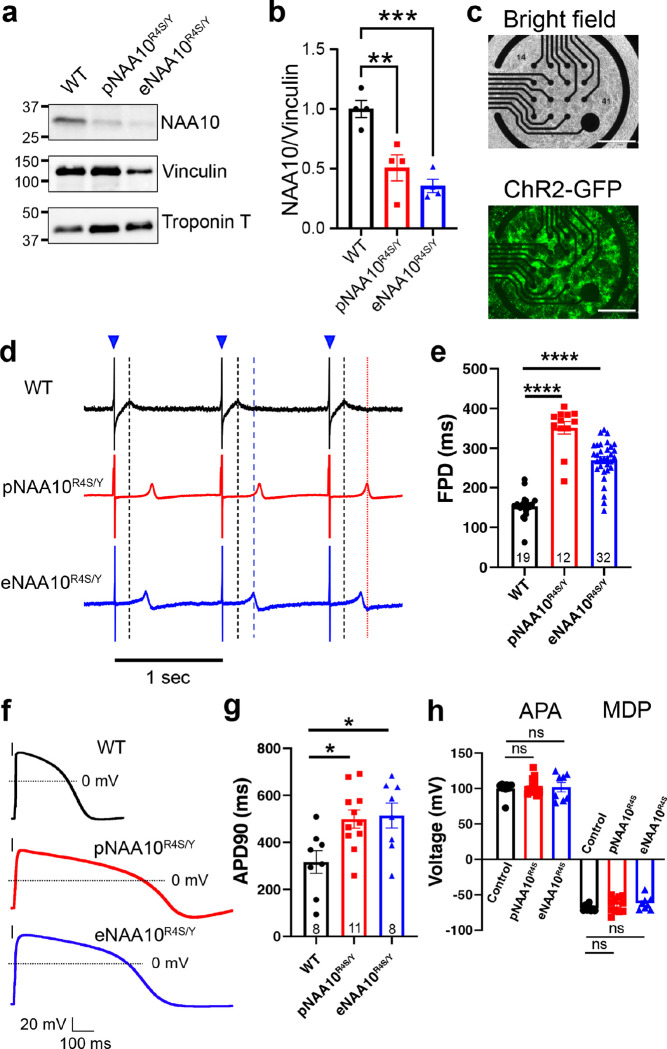
A cellular model of NAA10 dysfunction demonstrates repolarization abnormalities. **a.** Whole cell lysates from differentiated WT-, patient-derived (pNAA10^R4S/Y^)-, or genome-edited (eNAA10^R4S/Y^)-iPSC-CMs were separated by gel electrophoresis and subjected to Western blot analysis with antibodies specific to native NAA10, vinculin or cardiac troponin T. **b.** Quantification of total NAA10 protein levels were normalized to vinculin. **c.** Differentiated iPSC-CMs were plated on multi-electrode arrays (MEAs, upper panel) and transduced with adenovirus for the channelrhodopsin ChR2-GFP (lower panel) for optical pacing. Scale bar = 750μm **d.** Representative MEA recordings of WT-iPSC-CMs or harboring the NAA10 pR4S variant as either pNAA10^R4S/Y^- or eNAA10^R4S/Y^-iPSC-CMs with optical pacing at 1Hz with 488 nm light (blue triangles). Dashed lines represent the field potential duration (FPD) for WT-(black), pNAA10^R4S/Y^-(red), and eNAA10^R4S/Y^-(blue) iPSC-CMs. **e.** Quantification of FPD paced at 1Hz from B. FPD of pNAA10^R4S/Y^-, and eNAA10^R4S/Y^-iPSC-CMs. **f.** Representative whole-cell recordings of single iPSC-CMs under current clamp conditions, electrically paced at 1Hz. **g.** Quantification of the action potential duration (APD) at 90% of the peak membrane potential demonstrates significant prolongation in both pNAA10^R4S/Y^- and eNAA10^R4S/Y^-iPSC-CMs compared to control cells. **h**. The peak action potential amplitude (APA) and mean diastolic potential (MDP) were not affected by the NAA10^R4S^ variant. The number of analyzed samples (n) is annotated on each graph (sample numbers on panel g are the same for h), which were from at least three separate differentiations. Statistics were performed by the one-way Kruskal-Wallis test with Dunnet’s test for multiple comparisons: ns = p > 0.1, *p < 0.05, **p < 0.01, ***p < 0.005, ****p < 0.0001.

To model the effects of NAA10-R4S and gain mechanistic insight into NAA10 function in cardiomyocytes, we created induced pluripotent stem cell lines (iPSCs) from patients III:1 and III:8 through somatic-cell reprogramming^18^. Isolated iPSC clones were positive for the NAA10^R4S^ variant and had normal karyotypes and markers of pluripotency (Extended Data Fig. 3a-3c). A single patient-derived clone from patient III:8 was selected for further studies and was designated as pNAA10^R4S/Y^. To control for genetic background, we also introduced the NAA10^R4S^ variant into a male WT iPSC line by genome editing. This genome-edited isogenic line was designated as eNAA10^R4S/Y^ (Extended Data Fig. 3d). We also sequenced predicted off-target genome editing sites and did not detect any additional mutations (Extended Data Fig. 3e). We differentiated NAA10 mutant and WT-iPSC-lines into iPSC-CMs using established small molecule differentiation protocols^19^. We confirmed robust cardiomyocyte differentiation of all three iPSC lines by cardiac troponin T (cTnT) immunostaining followed by flow cytometry (Extended Data Fig. 3f). Western blotting of both pNAA10^R4S/Y^- and eNAA10^R4S/Y^-iPSC-CMs showed that the mutant lines expressed 50% less NAA10 protein compared to the control line (Fig. 3a, b), consistent with decreased stability of NAA10-R4S.

Prolonged repolarization is a hallmark of NAA10-related syndrome and the affected males in the NAA10-R4S pedigree. To determine if NAA10^R4S/Y^-iPSC-CMs recapitulate this phenotype, we used multi-electrode arrays (MEAs) to measure the field potential duration (FPD) of iPSC-CMs monolayers, which correlates with the ECG-based QT interval^20^. Since FPD depends on iPSC-CM beat rates^21^, we optically paced iPSC-CMs at a fixed rate by transducing them with adenovirus expressing the channelrhodopsin ChR2 fused to GFP (Ad-ChR2-GFP, Fig. 3c) and stimulating with 488 nm light at 1 Hz (Fig. 3d, blue triangles). Under these conditions, pNAA10^R4S/Y^- and eNAA10^R4S/Y^-iPSC-CMs had dramatically prolonged FPDs compared to WT-iPSC-CMs (WT = 150.5 ± 7.9 msec; pNAA10^R4S/Y^ = 349.7 ± 17.4 msec; eNAA10^R4S/Y^ = 268.3 ± 9.2 msec; P<0.0001; Fig. 3d and 3e).

To evaluate the effect of NAA10-R4S on cardiomyocyte repolarization more directly, we performed single-cell electrophysiology experiments on iPSC-CMs. After establishing a whole-cell current clamp configuration, we injected depolarizing currents at 1 Hz to elicit membrane action potentials (APs). iPSC-CM evoked APs had a characteristic ventricular-like morphology. The action potential duration (APD) measured at 90% of peak repolarization (APD90) was more than 2-fold longer in NAA10-mutant compared to WT iPSC-CMs (Fig. 3f,g). There were no differences in action potential amplitude (APA) or maximum diastolic potential (MDP) between WT- and NAA10^R4S/Y^-iPSC-CMs, suggesting the observed APD prolongation did not arise from differences in differentiation or maturation^22^. Collectively these data demonstrate that the NAA10^R4S^ variant causes severe repolarization abnormalities in iPSC-CMs.

### NAA10 dysfunction dysregulates both sodium and potassium currents.

To identify potential target proteins that might be affected by NAA10 dysregulation and cause repolarization abnormalities, we exploited the discovery that N-terminal protein sequence predicts the likelihood of Nt-acetylation. Therefore, we analyzed the N-terminal sequences of all proteins associated with cLQTS and their respective probability of modification by NatA (Table 1). This analysis suggested that multiple cardiac ion channels and related proteins known to influence cardiac repolarization could be NatA targets and therefore affected by NAA10 dysfunction. Among these candidates were genes that contribute to the voltage-gated sodium current (I_Na_; *SCN5A*), the slow activating potassium current (I_Ks_; *KCNQ1*), the rapid activating potassium current (I_Kr_; *KCNH2*), and the inward calcium current (I_Ca-L_; *CACNA1C*)^23^. We systematically evaluated the effect of NAA10^R4S^ on these currents.

**Figure 4:**
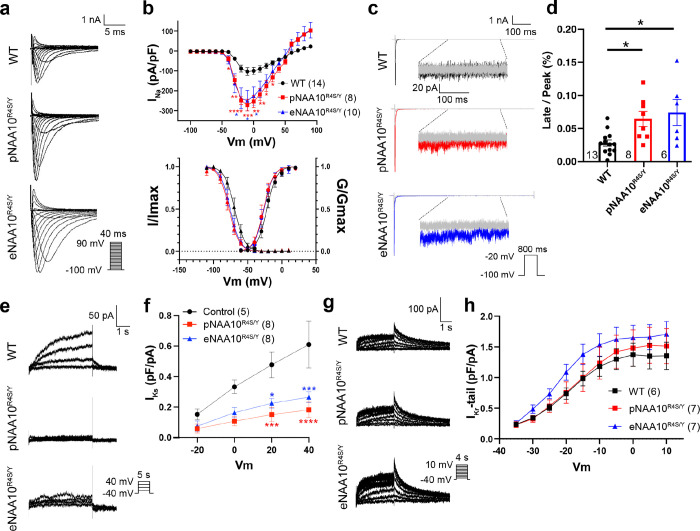
Increased late I_Na_ and decreased I_Ks_ contributes to action potential prolongation in NAA10-mutant iPSC-CMs **a.** Representative traces of sodium current (I_Na_) under whole-cell voltage-clamp conditions from WT-, pNAA10^R4S/Y^-, and eNAA10^R4S/Y^-iPSC-CMs. **b.** Current-voltage relation of normalized whole-cell I_Na_ with peak current densities significantly increased in both pNAA10^R4S^-, and eNAA10^R4S^-iPSC-CMs (upper panel). I_Na_ normalized with maximal conductance and maximal current density represent voltage-dependent activation and inactivation curves respectively (lower panel). **c.** Representative whole-cell current traces in response to long depolarizing pulses to elicit the persistent or late sodium current (I_NaL_) and normalized to tetrodotoxin blockade (TTX, gray traces). **d.** Quantification of I_NaL_ normalized to peak sodium current (I_NaP_) after leak subtraction with TTX. **e.** Representative whole-cell traces of the slow-rectifying potassium current (I_Ks_) from WT- and NAA10^R4S/Y^-iPSC-CMs. **f.** The I_Ks_ current-voltage relationship demonstrated decreased peak outward current densities in pNAA10^R4S/Y^-, and eNAA10^R4S/Y^-iPSC-CMs as compared to control cells. **g.** Representative whole-cell current traces of the rapid-activating potassium current (I_Kr_) in response to increasing voltage steps. **H.** After channel activation, the whole-cell voltage was stepped to the same voltage and the measured Instantaneous current was quantified as the I_Kr_-tail current for WT-, pNAA10^R4S/Y^-, and eNAA10^R4S/Y^-iPSC-CMs. For the voltage clamp recordings, the membrane capacitance of the cells was 49.8 ± 6.8, 46.8 ± 12.7, 43.7 ± 6.6 pF in WT-, pNAA10^R4S^-, and eNAA10^R4S^-iPSC-CMs, respectively. The number of cells (n) is annotated on each graph and samples were from at least three independent differentiations. Statistics were performed by one-way Kruskal-Wallis with Dunnett’s multiple comparisons test: ns = p > 0.1, *p < 0.05, **p < 0.01, ***p < 0.005, ****p < 0.0001.

SCN5A gain-of-function variants cause LQT type III (LQT3)^24^ through at least two mechanisms: (1) alterations in voltage dependent activation/inactivation and (2) increased late sodium current. To investigate the first mechanism, in the presence of the calcium channel blocker nifedipine (10 μM), depolarizing steps from a hyperpolarized holding potential of −100mV in single iPSC-CMs^25^ evoked stereotypical rapidly activating and deactivating currents consistent with I_Na_ (Fig. 4a). Peak inward sodium currents were significantly increased in pNAA10^R4S/Y^- and eNAA10^R4S/Y^-iPSC-CMs by as much as 2.5-fold compared to WT-iPSC-CMs (Fig. 4a and 4b upper panel). While increased I_Na_ current density is associated with heart failure and other forms of Na_V_1.5-mediated cardiovascular disorders, it is not a recognized mechanism for cLQTS. Instead, differential alterations in the voltage-dependent activation and inactivation of I_Na_ that increase the “window current” are associated with LQT3. While there was a small shift to more hyperpolarizing potentials in both the activation and inactivation curves in NAA10-mutant iPSC-CMs, there was no increase in the net activation probability (Fig. 4b lower panel, Extended Data Table 2).

*SCN5A* variants that increase the late sodium current (I_NaL_) also cause LQT3 and have also been associated with heart failure^26^. To investigate the effects of the NAA10^R4S^ variant and associated NAA10 dysfunction on I_NaL_, we stimulated iPSC-CMs with long-depolarizing steps (1s) at baseline and in the presence of 30 μM tetrodotoxin (TTX) to normalize for background membrane leak^27^. Consistent with a LQT3-type phenotype there was a > 2-fold increase in I_NaL_ in pNAA10^R4S/Y^- and eNAA10^R4S/Y^-iPSC-CMs as compared to WT-iPSC-CMs (Fig. 4c,d) suggesting a direct biophysical effect of NAA10 on Na_V_1.5 channel function.

*KCNQ1* variants that cause LQT1 reduce I_Ks_, while KCNH2 variants that cause LQT2 reduce I_Kr_. We isolated I_Ks_ by subtracting the current traces before and after administering the specific IKs blocker, HMR1556. (Fig. 4e). Activating voltage steps demonstrated a significant reduction in I_Ks_ current density in pNAA10^R4S/Y^- and eNAA10^R4S/Y^-iPSC-CMs within the physiologic depolarization range (Fig. 4e,f). Next, we elicited I_Kr_ by subtracting the current traces before and after the administration of specific I_Kr_ blocker, E4031. This maneuver did not reveal significant differences I_Kr_ in NAA10^R4S/Y^-iPSC-CMs compared to WT-iPSC-CMs (Fig. 4g,h, and Extended Data Fig. 4a). These data suggest that NAA10^R4S^ may affect KCNQ1 and reduce I_Ks_ repolarizing current.

CACNA1C variants that cause LQT8 affect the inward calcium (Ca^2+^) current I_Ca-L_^28^. We recorded L-type Ca^2+^ channels by inhibiting Na_V_1.5 channels with an elevating holding potential (−40 mV) in the recording bath. We did not observe significant differences in total current density or L-type Ca^2+^ channel properties in NAA10^R4S/Y^-iPSC-CMs (Extended Data Fig. 4c,d, Extended Data Table 1).

Taken together, these data indicate that QT prolongation and risk for arrhythmia in NAA10-related syndrome are caused by a combination of increased I_NaL_ and reduced I_Ks_.

### NAA10^R4S/Y^-iPSC-CMs have structural and contractile abnormalities.

**Figure 5:**
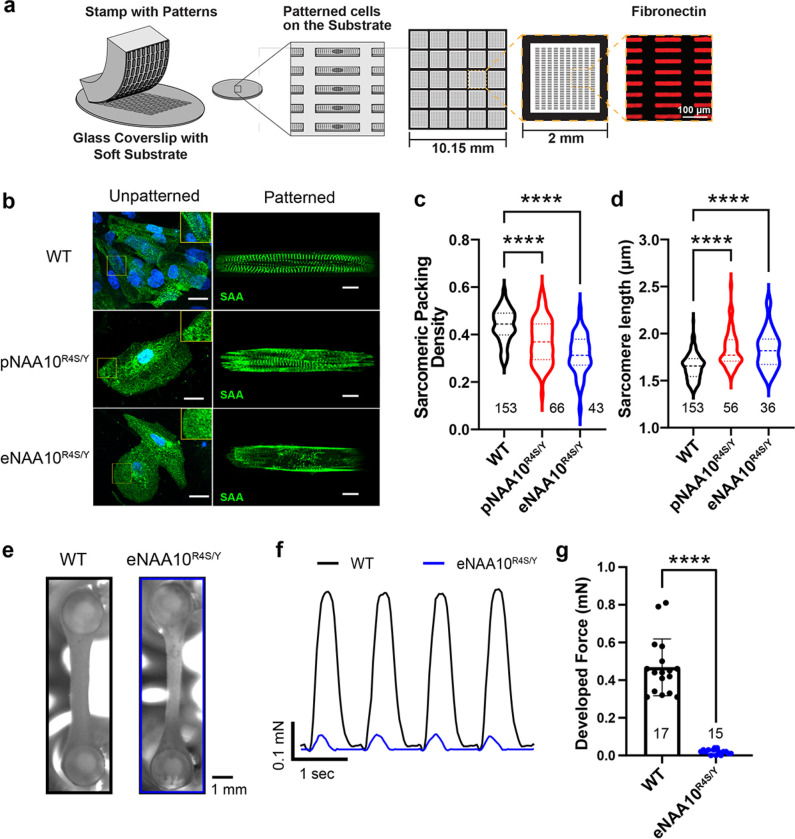
NAA10-mutant iPSC-CMs have severe structural and contractile defects. **a.** Depiction of micro-contact printing with PDMS stamps of fibronectin for spatially restricted patterning of iPSC-CMs. Rectangle patterns have a length to width ratio of 7:1. **b.** Confocal micrographs of WT-, pNAA10^R4S/Y^-, or eNAA10^R4S/Y^-iPSC-CMs, stained for sarcomeric alpha actinin (SAA) as either unpatterned (left panels, scale bar = 20μm) or patterned on micro-contacted printed substrates (right panels, scale bar = 10μm). **c.** A customized Matlab program calculated **C.** sarcomeric packing density (SPD) and **d.** sarcomere length from single micro-patterned iPSC-CMs. Lower SPD values correspond to a more disordered sarcomeric structure. **e.** Brightfield images of engineered heart tissues (EHTs) created from isogenic control (WT) and eNAA10^R4S/Y^-iPSC-CMs on PDMS “pillars’’ after transduction with ChR2-GFP adenovirus for optical pacing. **F.** Representative traces of developed contractile force of EHTs calculated from video data of PDMS pillar displacement in response to optical pacing with 488 nm light at 1Hz. **g.** Quantification of contractile force calculated over 10 beats from two independent differentiations. The number of cells or tissues (n) is annotated on each graph and samples were from at least three independent differentiations unless otherwise specified. Statistics were performed by one-way Kruskal-Wallis with Dunnett’s multiple comparisons test (c and d) or the Mann-Whitney test for g: ns = p > 0.1, *p < 0.05, **p < 0.01, ***p < 0.005, ****p < 0.0001.

Patients with NAA10-related syndrome have variable heart disease phenotypes ranging from hypertrophic cardiomyopathy to congenital heart disease^29^. Our patient III:8 died from complications related to severe dilated cardiomyopathy (DCM), which has not been previously associated with NAA10-related syndrome. To investigate possible cardiomyopathic effects of the *NAA10*^*R4S*^ variant and related NAA10 dysfunction, we analyzed the effect of NAA10 p.R4S on iPSC-CM structural assembly. We used micro-contact printing to deposit rectangular fibronectin islands with the 7:1 aspect ratio characteristic of adult human ventricular cardiomyocytes (Fig 5a)^30–32^. Plating iPSC-CMs on these patterned substrates induces sarcomere alignment, structural integrity, and features of cellular maturity when compared to unpatterned cells (Fig. 5b). High-resolution confocal imaging of iPSC-CMs stained for sarcomeric alpha actinin (SAA) and subjected to objective, computational image analysis^33^ demonstrated decreased sarcomeric packing density (SPD) and increased sarcomere length in NAA10^R4S/Y^-iPSC-CMs (Fig. 5c and 5d, respectively), consistent with a DCM-like phenotype^34^.

To further investigate the effects of NAA10 dysfunction on contractile force, we generated 3D-engineered heart tissues (EHTs) from WT and eNAA10^R4S/Y^-iPSC-CMs^35^. Cells embedded in extracellular matrix were molded around two silicone pillars and transduced with Ad-ChR2-GFP to facilitate optical pacing (Fig. 5e). After tissue formation and maturation, EHTs were optically paced at 1 Hz and imaged. Contractile force was measured based on pillar displacement (Fig. 5F). Despite long-term culture for up to 28 days, eNAA10^R4S/Y^-EHTs generated minimal force that was significantly lower than WT-EHTs (Fig. 5f,g). These data collectively identify novel structural defects and a severe contractile defect that was not observed in a previously reported model of NatA dysfunction caused by a NAA15^14^.

### Abnormal Ca^2+^ handling in NAA10^R4S/Y^-iPSC-CMs

**Figure 6:**
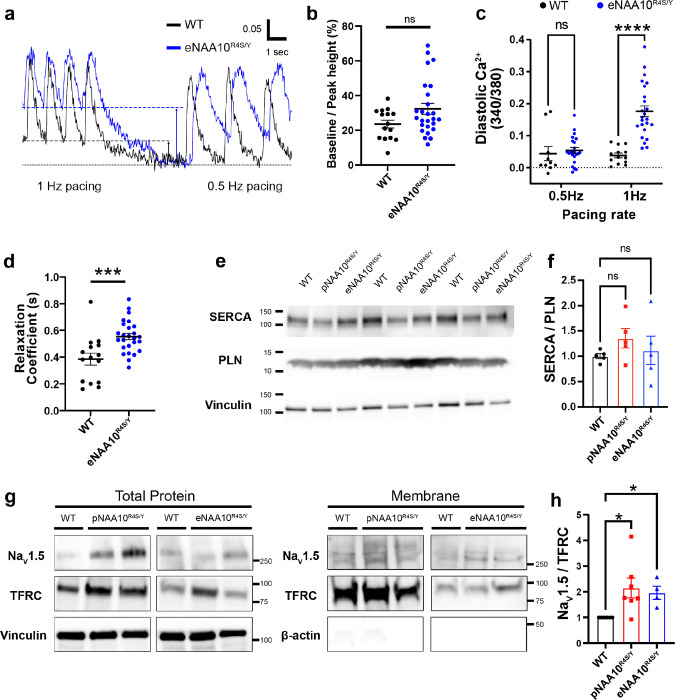
Alterations in Ca^2+^ and Na^+^ handling underlie NAA10^R4S/Y^ cardiomyocyte dysfunction. **A.** Micro-patterned single iPSC-CMs loaded with Fura-2 were electrically paced at 0.5 Hz and 1 Hz. Diastolic Ca^2+^ levels are indicated by dashed lines. **B.** Quantification of the base to peak height of Ca^2+^ transients paced at 0.5 Hz. **C.** End-diastolic Ca^2+^ levels were averaged for five transients and compared to the baseline with electrical pacing at 0.5 Hz and 1 Hz. **D.** Single exponentials were fit to the relaxation portion of each Ca^2+^ transient to calculate the relaxation coefficient **τ**. **E.** Whole cell lysates were analyzed by western blot with antibodies specific for SERCA2a (SERCA), phospholamban (PLN) and vinculin. **F.** The ratio of total PLN monomer to SERCA was quantified for each genotype. **G.** Cultures of iPSC-CMs were biotinylated and total and membrane fractions were isolated by streptavidin pull down. Western blot analysis confirms separation of total whole-cell lysates (left panel) and membrane fraction with the expression of the transferrin receptor (TFRC) and lack of beta-actin (right panel). **H.** The total membrane fraction of Na_V_1.5 was normalized to TFRC protein levels. Statistics by one-way ANOVA, **** p <0.0001, *** p <0.001, ns = > 0.05.

Balanced homeostasis of intracellular Ca^2+^ levels is critical for normal cardiomyocyte function. Indeed, excess diastolic Ca^2+^ is associated with impaired cardiomyocyte contraction and relaxation^36^. To determine if Ca^2+^ handling defects underlie impaired contractility in NAA10^R4S/Y^-iPSC-CMs, we used the ratiometric calcium indicator Fura-2 to record Ca^2+^ transients in patterned iPSC-CMs. With electrical pacing at 0.5 Hz, Ca^2+^ transients in eNAA10^R4S/Y^-iPSC-CMs did not have a significantly different amplitude or diastolic Ca^2+^ level (Fig. 6a–c). However, with pacing at a more physiologic rate of 1 Hz there was a significant increase in diastolic Ca^2+^ levels (Fig. 6a and 6b). Further, the calcium transient relaxation coefficient, a measure of the rate of cytosolic Ca^2+^ clearance, was significantly elevated in the eNAA10^R4S/Y^-iPSC-CMs compared to controls (Fig. 6a, 6d, and Extended Data Table 2).

During each Ca^2+^ transient, Ca^2+^ is cleared from the cytosol by re-uptake into the sarcoplasmic reticulum (SR) via the SR Ca-ATPase 2a (SERCA2a) and by Ca^2+^ efflux across the cell membrane via the sodium/calcium exchanger (NCX1)^36^. SERCA2a is inhibited by the binding phospholamban (PLN). To determine if SERCA2a or PLN levels were affected by NAA10 dysfunction, we performed western blotting on whole-cell lysates from WT-, pNAA10^R4S/Y^-, and eNAA10^R4S/Y^-iPSC-CMs. We did not observe significant changes in the level of these proteins or in the ratio of SERCA2a to PLN (Fig. 6e,f).

Next, we investigated non-SERCA Ca^2+^ efflux, which is influenced by cytoplasmic Na^+^ concentration. Our sodium channel recordings indicated increased activity in mutant NAA10^R4S/Y^-iPSC-CMs (Fig. 4a–d). To probe the underlying mechanism, we first determined if the NAA10^R4S^ variant affected SCN5A expression. Quantitative reverse transcription PCR did not reveal significantly altered levels of *SCN5A* or other ion channel transcripts in NAA10^R4S/Y^-iPSC-CMs (Extended Data Fig. 5). We next measured the surface expression of Na_V_1.5. After biotinylating surface-accessible iPSC-CM proteins, we then pulled down the membrane fraction with streptavidin. Western blot analysis revealed significantly greater surface expression of Na_V_1.5 in pNAA10^R4S/Y^- and eNAA10^R4S/Y^-iPSC-CMs (Figs. 6G and 6H), consistent with the higher I_Na_ density by whole-cell patch-clamp (Fig. 4a,b). These data suggest that NAA10 dysfunction increases Na_V_1.5 activity, inducing Na^+^ overload that increases diastolic Ca^2+^ levels via NCX1.

### Rescue of NAA10 disease phenotypes

**Figure 7:**
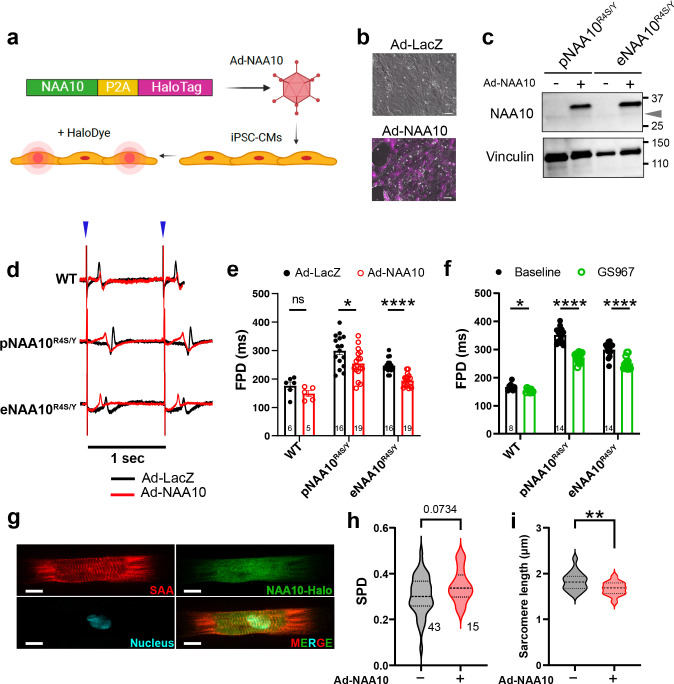
Genetic and pharmacologic rescue of NAA10-mutant iPSC-CMs. **a.** Expression vectors for LacZ or NAA10-P2A-HaloTag were packaged into adenovirus and used to transduce monolayers of iPSC-CMs. **b.** Incubation with HaloTag dye indicates expression of NAA10 (lower panel). Scale bars = 100 μm. **c.** Western blot analysis of whole-cell lysates from pNAA10^R4S/Y^- or eNAA10^R4S/Y^-transduced with Ad-LacZ or Ad-NAA10 and probed with anti-NAA10. The faint lower band represents native NAA10 expression (gray triangle). **d.** Representative MEA recordings of monolayers of iPSC-CMs co-transduced with Ad-ChR2-GFP and either Ad-LacZ or Ad-NAA10, plated on MEAs and optically paced at 1 Hz (blue triangles). **e.** Quantification of field potential duration (FPD) from D measured as 48 hours after adenovirus transduction. **f.** Monolayers plated on MEAs were first measured at baseline and then treated with the INaL blocker GS967. The absolute FPD was determined for each genotype and compared as paired treatments. **g.** Single micro-patterned iPSC-CMs transduced with Ad-NAA10 were stained for SAA to quantify changes in sarcomeric organization. Automated analysis of single iPSC-CMs on micro-patterned substrates for **H.** sarcomeric packing density and I. Sarcomere length.

In our NAA10-related syndrome iPSC models, decreased NAA10 protein level and catalytic activity correlated with cardiomyocyte dysfunction, suggesting that restoration of NAA10 activity may reverse the disease phenotypes. To test this hypothesis, we generated adenovirus that expresses WT NAA10 along with the self-labeling protein HaloTag (Ad-NAA10, Fig. 7a). We transduced cultures of NAA10^R4S/Y^-iPSC-CMs with either Ad-NAA10 or control adenovirus expressing LacZ (Ad-LacZ) (Fig. 7b). Western blot analysis of iPSC-CM lysates 48 hours after transduction demonstrated robust expression over baseline levels (Fig. 7c). The endogenous protein migrated at a different size than exogenously expressed NAA10 (Fig. 7c, gray arrow).

Next, we characterized the effect of Ad-NAA10 on NAA10-R4S mutant and wild-type iPSC-CMs. We transduced iPSC-CM monolayers plated on MEAs with either Ad-LacZ or Ad-NAA10. Cells were also transduced with Ad-ChR2-GFP. Under optical pacing at 1 Hz, Ad-NAA10 did not significantly affect wild-type iPSC-CMs, suggesting that NAA10 overexpression is well tolerated (Fig. 7d,e). Meanwhile, Ad-NAA10 significantly shortened FPD in NAA10-R4S mutant iPSC-CMs (Fig. 7d,e).

To determine if NAA10 over-expression reverses the observed structural defects in NAA10^R4S/Y^-iPSC-CMs, we transduced iPSC-CMs with Ad-NAA10 and plated them on micro-contact printed substrates (Fig. 7g). Following immunostaining and confocal imaging, unbiased computational image analysis demonstrated significant shortening of the mean sarcomere length (Fig. 7i) and a trend towards increased sarcomeric packing density (Fig. 7h), suggesting a partial rescue of structural phenotypes by NAA10 supplement. Collectively, these data demonstrate that gene replacement may be a viable therapeutic strategy for NAA10-related syndrome.

To assess the contribution of increased I_NaL_ to the repolarization abnormalities in pNAA10^R4S/Y^- or eNAA10^R4S/Y^-iPSC-CMs, we treated MEA-plated monolayers with the selective late current sodium channel blockers GS967 and ranolazine^27^. GS967 and ranolazine significantly shortened FPDs of NAA10^R4S/Y^-iPSC-CMs but did not completely normalize them to WT values (Fig. 7f; Extended Data Fig. 6). In contrast, mexiletine, a class IB antiarrhythmic which also has effects on potassium channels^37^, did not significantly change the FPD (Extended Data Fig. 6).

## Discussion

In this study we gained new mechanistic insights into the function of NAA10 in cardiac homeostasis. Capitalizing on the identification of a novel NAA10 variant in an extended pedigree, we created and characterized an iPSC-CM model of NAA10 dysfunction to show that Nt-acetylation by NAA10 is required for normal regulation of I_NaL_ and I_Ks_, diastolic Ca^2+^, and for normal sarcomere assembly and contraction.

From our clinical cohort, we identified a novel NAA10 variant that segregated with disease phenotype supporting its pathogenicity. A KCNH2 variant was also present in some family members, but it did not co-segregate with the cardiac phenotypes and there is little evidence that it is pathogenic. Consistent with other reports of NAA10-related syndrome, we observed variable clinical manifestations in gene-positive patients, with a predominance of SCD in male patients. However, at least one terminal cardiac event occurred in a carrier female (III:6). One of our patients also developed severe dilated cardiomyopathy. Combined with our experimental data, these patients broaden the potential cardiac phenotypes associated with NAA10-related syndrome, although confirmation requires study of additional affected patients.

Like other NAA10 syndrome patients with cardiac involvement, our identified variant is near the extreme N-terminus of NAA10 and within the NAA15 interaction and catalytic domains. Our biochemical data demonstrates that the p.R4S mutation not only induces NAA10 protein degradation and directly inhibits its Nt-acetylation activity but also destabilizes NAA10’s interaction with NAA15. Our results parallel an early report of NAA10-related syndrome, where the pathogenic variant p.Y43S induced NAA10 protein degradation and impaired catalytic function^8^. However, the authors did not directly investigate the effects of the Y43S variant on the interaction of NAA10 and NAA15, nor did they study the impact of NAA10 dysfunction on cellular physiology. Our data show that the NAA10 p.R4S is a pathogenic variant, delineate molecular mechanisms that lead to NAA10 dysfunction, and delineate the consequences of NAA10 dysfunction on cardiomyocyte physiology.

To investigate the effects of NAA10 dysfunction, we developed the first iPSC models through somatic-cell reprogramming from affected patients and created an isogenic iPSC line by introducing the same variant into a control line by genome-editing. Our iPSC lines are accurate models of NAA10 dysfunction based on several lines of evidence. First, NAA10 protein levels in mutant iPSC-CMs are reduced by more than 50% consistent with reduced protein stability. Second, NAA10^R4S/Y^-iPSC-CMs have repolarization abnormalities as both monolayers and single cells consistent with QT prolongation. Third, single iPSC-CMs and EHTs derived from NAA10^R4S/Y^ lines had profound defects in sarcomere formation and contractility respectively. These data strongly support that our NAA10-mutant iPSC models accurately recapitulate the clinical phenotype of severe arrhythmogenesis, and cardiomyopathy as seen in our clinical cohort.

Clinical data and our experimental data indicate that NAA10 is required for normal cardiac repolarization. To define the physiological mechanisms, we systematically examined each major ionic current underlying the cardiac action potential and found that NAA10 dysfunction alters both I_Na_ and I_Ks_. This unique combination induces significant repolarization abnormalities consistent with a high-risk clinical phenotype (QT > 500msec, recurrent TdP) and similar to patients with complex heterozygosity and missense variants in both *KCNQ1* and *SCN5A*^38^. The male NAA10 p.R4S patients had ventricular arrhythmias predominantly during periods of rest and despite high dose beta-blocker therapy. These are typical clinical features of pathogenic SCN5A variants and LQT3, and support an important role for increased I_NaL_ in abnormal repolarization in this disorder. This is further supported by the shortening of the FPD in response to GS967 and ranolazine, both selective INaL blockers^27,39^. In contrast mexiletine, a class 1B sodium channel blocker, failed to shorten the FPD in MEA recordings of eNAA10^R4S/Y^-iPSC-CMs. In addition to inhibiting I_NaL_, mexiletine also inhibits IKr and can prolong the APD in iPSC-CMs^40^. Since NAA10 dysfunction impairs the remaining repolarizing potassium current, I_Ks_, mexilitine’s effect on I_Kr_ likely accounts for its ineffectiveness in normalizing repolarization of NAA10^R4S/Y^-iPSC-CMs. Removal of mexiletine’s off-target effects against I_Kr_, has been the subject of recent research to improve mexiletine as a selective therapy for LQT3 patients^40^, and our data suggest that this selectivity will also benefit NAA10-syndrome patients.

Our data also point to an important role for NAA10 in cardiomyocyte sarcomere assembly and contractile function. In general, monogenic causes of cLQTS are not associated with decreased contractility or heart failure^28^. A potential exception to this convention is that selected SCN5A variants are associated with DCM and QT prolongation in some patients^41^. We likewise observed increased I_NaL_ in our iPSC models of NAA10 dysfunction. The resulting increased sodium flux could contribute to NAA10-mediated contractile dysfunction by elevating diastolic Ca^2+^ levels through increased activity of the sodium calcium exchanger. We also observed abnormal sarcomere structure in NAA10 mutant iPSC-CMs, indicating that contractile dysfunction in this disorder is likely multifactorial.

Our iPSC lines represent the first models of NAA10 dysfunction demonstrating both repolarization and severe contractile abnormalities. A previous report by Ward et. al. modeled NAA15 dysfunction in iPSC-CMs but only detected minor contractile impairment^14^. This could represent incomplete inhibition of the NatA complex, or differential targets caused by individual subunit dysregulation. There was no electrophysiologic assessment of NAA15-mutant iPSC-CMs, precluding comparison of the effects of NAA15 versus NAA10 dysfunction on the cardiomyocyte action potential. However, given that NAA10-R4S affects both NAA10 function and its association with NAA15, effect severity may correlate with a hierarchy of NatA complex formation > NAA10 function > NAA15 function. Further investigation is necessary to understand the effect of distinct NAA10 or NAA15 variants on cardiomyocyte function. iPSC disease modeling has dramatically shortened the arc of therapeutic translation. The repurposing of already approved drugs^42^ and development of targeted gene therapies are just two therapeutic development pathways that have benefited from iPSC modeling of cardiac disorders^50^. To determine if NAA10-related syndrome would be amenable to targeted gene therapy, we developed an adenovirus vector to over-express WT NAA10 in the NAA10-mutant iPSC-CMs. This therapy partially rescued key pathogenic features of NAA10 dysfunction. Incomplete rescue may be due to transduction efficiency, NAA10 expression level, and dominant negative effects of the mutant endogenous allele. The short duration of our experiment compared to the turnover rate of key Nt-acetylation targets could have also limited the therapeutic effect, given that Nt-acetylation is a permanent co-translational PTM. Further optimization and testing in animal models of NAA10 dysfunction will be critical for effective translation.

Our detailed physiological characterization of the first iPSC models of NAA10 dysfunction define essential roles of protein Nt-acetylation in cardiomyocyte homeostasis and delineate the pathogenic mechanisms by which NAA10 dysfunction leads to QT prolongation, risk for sudden death, and contractile dysfunction.

## Online Methods

### Patient Data

The proband was identified as part of a large family originally diagnosed with “gene-negative” long QT syndrome. Retrospective clinical data was collected after enrollment into an Institutional Review Board (IRB) approved protocol at Boston Children’s Hospital. All patient-related information was contained in an encrypted password-protected database (RedCap) with only de-identified samples available to researchers. A four-generation family history was performed by a certified genetic counselor. Initial genetic testing for Long QT syndrome included: *CACNA1C, CALM1, CALM2, CALM3, KCNE1, KCNH2, KCNJ2, KCNQ1, SCN5A, TRDN, AKAP9, ANK2, CAV3, KCNE2, KCNJ5, SCN4B*, and *SNTA1* (Invitae, test code 02211). Testing for cardiomyopathies included up to 121 genes (Invitae, test code 02251) with the NAA10-R4S variant identified on the neurodevelopmental disorders panel (Invitae, test code 728434). Echocardiography, electrocardiography, and remote device monitoring were performed as standard of care at Boston Children’s Hospital and Dartmouth Hitchcock Medical Center. Postmortem examination with cardiac histopathological evaluation was performed by the Cardiac Registry service at Boston Children’s Hospital.

### Generation of iPSC lines

Patients who consented to participate in this study supplied peripheral blood mononuclear cells (PBMCs) for somatic-cell reprogramming into iPSCs. This protocol was IRB approved at Boston Children’s Hospital and participation was offered to patients with NAA10 variants as part of a larger study on inherited arrhythmia syndromes. Patients were recruited through electrophysiology clinics at both Boston Children’s Hospital and Dartmouth Hitchcock Medical center. Consent was obtained by the study coordinator. Variant sequencing, pluripotency marker staining, and karyotype analysis performed at regular intervals ensured iPSC model integrity.

### Gene-editing of iPSCs

For genome-editing, variant specific sgRNAs were designed with CRISPOR^43^, and synthesized using EnGen sgRNA Synthesis Kit (E3322; New England Biolabs, Ipswich, MA, USA). HDR donor template (Integrated DNA Technologies, Coralville, IA, USA) was designed to include synonymous mutations to avoid further digestion by Cas9.

A doxycycline-inducible WT iPSC-line (WTC-Cas9)^48^, which was previously generated from a wild-type human male iPSC line (Coriell Institute: # GM25256), was used for gene-editing as previously described^44^. Briefly, 16 hours before nucleofection, doxycycline was administered at the final concentration of 2 μg/mL. 5 μg of sgRNA and 5 μg of HDR template were transfected into 1 × 10^6^ doxycycline-treated WTC-Cas9 iPSCs using Human Stem Cell Nucleofector Kit (VPH-5012; Lonza Bioscience, Basel, Switzerland) and 4D-Nucleofector (Lonza Bioscience). The next day, the medium was replenished with fresh mTeSR1 without doxycycline, and the single cells were seeded sparsely to a 10cm dish a few days later. Thereafter, colonies were picked up for sequencing. Off-target sites were predicted by CRISPOR^43,45^, and the sequence was analyzed by Sangar sequencing.

### Differentiation and purification of iPSC-CMs

iPSCs were differentiated into CMs as previously described^46^. Briefly, 2 days after iPSCs were seeded onto a 12-well plate, the medium was replaced with RPMI / B27 minus insulin (Thermo Fisher Scientific, Waltham, MA, USA) containing 6 μM CHIR99021 (Stemcell technologies, Vancouver, Canada) (Day 0). On Day 2, the medium was replaced with RPMI/B27 minus insulin containing 5 μM IWP2 (Tocris Bioscience, Bristol, UK). On Day 4, the medium was replaced with RPMI/B27 minus insulin. On Day 6 and every 3 to 4 days thereafter, RPMI/B27 medium was replenished. CMs were purified in glucose-depleted lactate-supplemented medium as previously described^47^. Differentiated CMs on Days 50 through 90 were used for experiments.

For large scale differentiation, iPSCs were differentiated in the DASGIP^®^ Parallel Bioreactor Systems (Eppendorf) as described previously^49^ with several modifications that will be part of an independent manuscript. In short, iPSCs were cultured in T80 cell culture flasks (Life Technologies, # 178905) pretreated with 1:100 (v/v) diluted Geltrex (Life Technologies, # A1413302) at an initial seeding density of 15,000 cells/cm^2^. Cells were maintained in E8 medium (Life Technologies, # A1517001) with daily medium change until 90% cell confluency was reached. For dissociation, T80 flasks were washed once with phosphate buffered saline (PBS), incubated with 5 mL Versene (Life Technologies, # 1540066) for 15 to 20 min at 37°C and dissociation was stopped by adding 5 mL E8 medium. 50 million single iPSCs were resuspended in 50 mL E8 medium supplemented with 10 μM of ROCK inhibitor. The bioreactor vessel was taken from the bioreactor system and placed under the laminar flow and 50 mL E8 (+ 10 μM Y-27632) and 50 mL of the iPSC solution resulting in a final volume of 100 mL per vessel. Cells were agitated at a speed of 60 rpm, passed with 21% O_2_ and 5% CO_2_ by 10 sL/h overlay gassing and maintained at 37°C. The next day diameter of spontaneously formed embryoid bodies (EBs) was measured to estimate time of differentiation start. If critical diameter (100 – 300 μm) was reached, cardiac differentiation was induced by a complete change of the medium to RPMI 1640 with B27 supplemented with 7 μM CHIR99021 (day 0). After 24 h (Day 1), the complete medium was changed to basic medium, and cells were incubated for an additional 24 h. On day 2, the complete medium was changed again to basic medium containing 5 μM IWR-1-endo for 48 h. On day 4, the complete medium was changed to basic medium, and cells were incubated for an additional 72 h. From day 7 on cells were cultured in basic medium supplemented with 1:1000 (v/v) insulin (Sigma-Aldrich, # I9278) followed by 50% medium refreshments at day 9, 11 and 13. Finally, cells were dissociated on day 15 for 3–4 h depending on EB size and density using Collagenase II (Worthington, # LS004176)^35^, and frozen using a controlled rate freezer (Grant, CRF-1).

Patient-derived and genome-edited iPSC lines may be obtained upon reasonable request with an appropriate Materials Transfer Agreement and corresponding Institutional Review Board Protocol in place at the requesting academic institution. Stem cell lines will be provided but no identifiable patient data will be supplied with the patient lines. An experimental plan, timeline, and confirmation that if whole-genome sequencing is performed, the providing institution is notified and the data is reviewed prior to publication or analysis by any 3^rd^ party. No patient data or iPSC lines may be transferred to or sold to any commercial party without the express permission of the providing institution, Boston Children’s Hospital, and the department of Cardiology.

### Electrophysiology

#### Whole-cell patch clamp recordings

Cultured iPSC-CMs were dissociated with Accutase and plated sparsely onto Geltrex-coated 11mm coverslips. Single iPSC-CMs were analyzed 3 to 6 days after dissociating. Single iPSC-CMs were recorded under different conditions to acquire each parameter (Supplemental Table 1)^50,51^. Perforated patch recordings were performed for action potential (AP) analysis and the L-type Ca^2+^ current (I_CaL_). Perforated patch was applied in order to prevent run-down in I_CaL_ recording^52^. The ruptured patch technique was used for I_Na_, I_Ks_, and I_Kr_ recordings. Series resistance and cell capacitance were compensated to ~ 60 % for all the voltage clamp experiments. To measure I_Na_, starting from a holding potential of −100 mV, 40 ms of depolarizing pulses from −100 mV to 90 mV were applied in 10-mV increments. For the I_Na_ steady-state inactivation, following 400 ms of prepulses with 10-mV increments from −110 mV to −20mV, 40 ms of 0 mV pulse was applied. For I_Ks_, test pulses were applied for 5 s with 20 mV increments from −20 mV to 40 mV from a holding potential of −40 mV. For I_Kr_, test pulses were applied for 4 s with 5 mV increments from −35 mV to 10 mV from a holding potential of −40 mV. To measure I_CaL_, starting from a holding potential of −80 mV, 3-s long −50mV prepulse was applied and then 300 ms of depolarizing pulses from −50 mV to 50 mV were applied in 10-mV increments. For the I_CaL_ steady-state inactivation, from −40mV holding potential, test pulses were applied for 2s with 10 mV increments ranging from −80mV to 10mV followed by 10-ms-long −40mV pulse, and then 0mV pulse was applied for 250ms. For AP analysis, iPSC-CMs exhibiting a APD90 / APD50 ratio of less than 1.4 were defined as ventricular type^53^. I_NaL_, I_Ks_, and I_Kr_ were defined as currents specifically sensitive to 30 μM TTX, 1 μM HMR1556, and 1 μM E4031 respectively. The current traces were subtracted before and after the drug administration to elicit those specific currents. Pipettes were pulled from thick-walled borosilicate glass capillaries (1B150F-4; World Precision Instruments, FL, USA) for AP and I_CaL_, and from thin-walled capillaries (TW150–4; World Precision Instruments) for I_Na_, I_Ks_, and I_Kr_. The resistance of the pipettes for INa recording was 1 – 2 MΩ. For the other recordings, the pipettes with 3 – 5 MΩ were used. β-escin (25 μM) was applied in the recording solution to create the perforated patch configuration^54^. Access resistance was 10 – 25 MΩ for perforated patch recording and < 5 MΩ for ruptured patch recording. dPatch^®^, and SutterPatch^®^ (Sutter Instrument, CA, USA) were used for data acquisition. All the data were acquired from at least three independent experiments using different biological replicates.

### Multi-electrode array with optogenetics

Single iPSC-CMs were isolated by incubating collagenase-B (Roche, Roswell, GA, USA, 1mg/mL) for 15 minutes and thereafter 0.25% Trypsin or Accutmax (Innova Cell Technologies, San Diego, CA, USA) for 5 minutes for the dissociation as previously described^53^. Cell suspensions (3 × 10^4^ cells in 5 μL) were placed onto fibronectin-coated multi-electrode array (MEA) plates (CytoView MEA; Axion BioSystems, Atlanta, GA, USA). After 3 days, the CMs were infected with the crude adenovirus expressing Channelrhodopsin-2 fused to green fluorescent protein (GFP) (Ad-ChR2-GFP) to enable optical stimulation^55^. Four or more days after the infection, field potentials (FP) were recorded using Maestro Edge (Axion BioSystems). FP signals were digitally sampled at 12.5 kHz and the system bandwidth is 0.01 Hz – 5 kHz. iPSC-CMs were stimulated by using a multi-well light stimulation system (Lumos 24; Axion BioSystems). Specifically, CMs were stimulated at the rate of interest (1 Hz, 2 Hz, or 3 Hz) for 40 beats and the final 30 beats were averaged. FP duration (FPD) was defined as the interval between a spike and a subsequent positive deviation. This parameter was automatically measured with Cardiac Software Module on the system. All the data were acquired from at least three independent biological replicates.

### Immunofluorescence

Samples were washed with cold Ca^2+^-free PBS for 5 minutes before fixed with 4% paraformaldehyde for 10 minutes at 4°C, and permeabilized with 0.1% Triton X in PBS for 10 minutes at room temperature. Blocking was performed with 3% bovine serum albumin in PBS. Primary antibodies and secondary antibodies were sequentially incubated for 2 hours at room temperature, followed by serial washes with PBS and DAPI in the mounting media. The details of antibodies are listed in supplemental table. The coverslips with stained samples were mounted with Prolong Diamond Antifade Mount (Invitrogen, 2273639), and after 24 hours of incubation in dark conditions at room temperature, imaging was performed. Confocal microscopy (Olympus FV3000R) with a 60x oil immersion objective was used.

### Micro-contact patterning and sarcomere analysis

Using Computer-Aided Design (CAD), the single-cell pattern was first designed as a series of rectangles with a 7:1 aspect ratio (105 μm by 15 μm) surrounded by a 220 μm thick boundary. The design was then converted to photolithography masks (CAD/Art Services Inc.). At the Center for Nanoscale Systems (Harvard University), silicon wafers (Wafer World) with a diameter of 3 inches are cleaned with a nitrogen gun and then spin coated with photoresist SU8–3005 (MicroChem Corp.), followed by cycles of 1 minute and 2 minutes of baking at 65 and 95°C, respectively. Polymerization via UV-light exposure through the photomasks with the desired single-cell patterns was then executed for 20 seconds. The cycles of baking were repeated, and the post-UV wafers were developed in propylene glycol methyl ether acetate (PGMEA, Sigma) for no more than 1 minute under vigorous agitation. After development, the wafer was desiccated overnight with a small amount of silane (United Chemical) to prevent Polydimethylsiloxane (PDMS) from binding to it permanently. Once the wafers were prepared, Polydimethylsiloxane (PDMS, Sylgard 184; Dow Corning) prepared at a ratio of 10:1 (base: curing agent) was placed on the wafer, covering its entire surface, and baked for 24 hours at 65°C. The next day, stamps were cut without damaging the wafer and sonicated in 70% ethanol for use in patterning.

Glass coverslips (12 mm, VWR, 48366–252) were spin-coated at custom recipes with a 1:1 ratio of Polydimethylsiloxane (PDMS) elastomer (Sylgard 184, The Dow Chemical Company, Midland, MI, USA) and dielectric gel (Sylgard 527, The Dow Chemical Company). The latter, PDMS 527, was prepared via combining Part A and Part B of the kit in a 1:1 ratio. Coverslips were coated for 48 hours in a 65°C oven. Thereafter, stamps containing rectangular-shaped islands with 7:1 aspect ratio^30^ were first coated for 1 hour with Fibronectin (Sigma Aldrich F0895) (50 μg/ml) diluted in Geltrex (1:200, Life Technologies, A1413302). Fibronectin aliquots of 1 mg/ml were prepared in PBS and stored at −20°C. In the meantime, PDMS-coated coverslips are exposed to UV Ozone (Jelight) for 8 minutes and the patterning process was performed by placing the dried stamps onto the coverslips. The coverslips were immersed in 1% Pluronic F-127 (Sigma-Aldrich, P2443) for less than 10 minutes to block the portions of the coverslips not coated with fibronectin, followed by washing 3 times with room-temperature PBS.

iPSC-CMs were seeded onto the micropatterned coverslips 3 days before immunofluorescence imaging. Cells were stained with FITC-conjugated α-sarcomeric actinin, and Alexa-647 conjugated Phalloidin, followed by staining with Hoechst 33342.

To assess sarcomere alignment in micropatterned iPSC-CMs, we used an unbiased algorithm developed by the Disease Biophysics Group incorporating the ImageJ Plugin (Orientation J) and a custom-made MATLAB (Mathworks) script for structural analysis of single cells^33^. Briefly, a Sarcomere Packing Density (SPD) reflects the degree of spatial organization of the sarcomeres quantifying the immunosignal localized in a regular lattice and the periodicity of the positive structures respectively of their orientations. This means that the poorly formed sarcomeres that are not periodically spaced demonstrating a reduced SPD value, ranging from 1 to 0.

### EHT generation

Engineered heart tissues (EHTs) were generated as described previously^35,56^ with some minor modifications. Briefly, 3D-differentiated 0.8×10^6^ hiPSC-CMs were used to generate each EHT. Cells were transduced with Ad-ChR2-GFP on the day of casting or after 7 days in vitro. We modified the standard EHT culture medium (EHT-medium in the referenced literature^35^) by replacing DMEM with RPMI 1640 plus B27 minus insulin, removing 10% heat-inactivated horse serum, and reducing aprotinin concentration to 5 μg / ml. EHT contraction was recorded as described below from day 7 on and functional analysis was performed from day 27 to day 33.

### Functional assessment of EHTs

EHTs in a 24 well plate were placed in a stage top incubator and maintained at 37 C, 5% CO2. EHTs were optically paced at different frequencies using blue LEDs positioned above the place and recorded from below at 30 frames per second through a 561 nm long-pass filter (Semrock BLP02–561R-32) using an 8mm f/1.4 lens (ThorLabs MVL8M1) mounted on a Basler acA1920 camera. EHT post movement was tracked post-hoc using the multi-template matching FIJI plugin and subsequently analyzed using a custom Python script^57^. Twitch force measurements were subsequently measured by applying post deflection to the beam bending theory for a known Young’s modulus of the posts, as described in detail elsewhere^58^. The custom Python code will be available on GitHub with appropriate version control.

### Quantitative PCR

Cells were washed once with ice-cold PBS and lysed in TRIzol. Total RNA was extracted by centrifugation and RNA samples were isolated. Coding DNA (cDNA) was made using a reverse transcriptase kit (Superscript III, Invitrogen). We quantified total cDNA for each sample and normalized the concentration. Quantitative PCR was performed on a 96 well thermocycler (BioRad) at an annealing temperature of 55°C with validated gene-specific primers. Ct values were compared to a house-keeping gene (GAPDH) and the fold-change was calculated and compared to control samples for each gene transcript.

### Western blot

Cells were lysed with mTOR lysis buffer (120mM NaCl, 40mM HEPES, 40mM NaF, 1mM EDTA, 10mM β-Glycerophosphate disodium, 0.3% CHAPS, pH 7.5 with NaOH) containing 1% TritonX and Halt protease and phosphatase inhibitor (Life Technologies 78442). The concentration of the protein was measured with BCA protein Assay kit (Thermo scientific 23225) and 3 μg of protein in each lane was analyzed by SDS-PAGE and immunoblotting. Blots were incubated with primary antibodies and secondary antibodies sequentially for 2 hours at room temperature or overnight at 4°C. Protein signals were detected using an enhanced chemiluminescent substrate (BioRad), and images were captured using Azure 300 (Azure Biosystems, Dublin, CA, USA) and analyzed with ImageJ. The antibodies are listed in the supplemental table.

### Expression plasmids

NAA10^WT^-3×FLAG cloned into pUC-GW-Kan vector was synthesized by a manufacturer (GENEWIZ, South Plainfield, NJ, USA). Then, NAA10 ^WT^ −3xFLAG was cloned into pcDNA3.1(+) vector, and NAA10^R4S^-3xFLAG/pcDNA3.1(+) was generated by site-directed mutagenesis with In-Fusion cloning (Takara Bio, Kusatsu, Japan).

NAA15 expression plasmid (HG19640-UT, Sino Biological) was also cloned into pcDNA3.1(+) vector and myc tag was added using In-Fusion cloning.

### Cycloheximide chase experiment

2.5 μg of plasmids were transfected into HEK293T cells on a 6-well plate with Lipofectamine 3000. 48 hours after the transfection, the medium of each well was replaced with 2 ml culture medium containing 50 μg/mL Cycloheximide (Sigma, 01810). Cells were harvested at 0, 2, 4, and 6 hours after the Cycloheximide administration. The cells harvested at 0 hour were not treated with Cycloheximide. Harvested cells were centrifuged at 4°C for 15 seconds with 14000 g, and the cell pellets were washed with ice-cold PBS, centrifuged again, and stored at −80°C after the supernatant was discarded. After all the samples were harvested, western blot was performed, and the membrane was stained with anti-DDDDK tag antibody (Abcam, ab1257) and anti-Vinculin antibody (Santa Cruz). For secondary antibodies, IRDye680 RD donkey anti-goat (LI-COR Biosciences, Lincoln, NE, USA) and IRDye800 CW donkey anti-mouse (LI-COR Biosciences) were used. Imaging was performed with LI-COR Odyssey Infrared Imaging System (LI-COR Biosciences).

### NAA10-NAA15 binding assay

The NAA10- and NAA15-expression plasmid was transfected to HEK293 cells with Lipofectamine 3000. 48 hours later, the cells were harvested and lysed with mTOR lysis buffer. The lysate was incubated with anti-FLAG magnetic beads overnight at 4°C. The protein was eluted with FLAG peptide and analyzed with SDS-PAGE. Immunoblotting was performed as described above. The protein was quantified using ImageJ.

### Protein synthesis

50 μg of the NAA10 expression plasmid was transfected into HEK293 cells on a 15 cm dish using polyethylenimine (PEI) (Sigma 408727). 4 dishes were prepared for each plasmid, and after lysing the cells the cell lysate was filtered with a 0.22μm filter, and pre-cleared with mouse IgG agarose. Thereafter, FLAG-tagged NAA10 was pulled down with FLAG tag using anti-FLAG magnetic beads (Sigma M8823). The protein was eluted with FLAG peptide and concentrated using Amicon Ultra-4 Centrifugal Filter Unit (Millipore, UFC8010).

### ThioGlo4 assay

ThioGlo4 assay was modified from a previous protocol^59^. The custom peptide as a substrate for NAA10 (EEEIA24: EEEIAALRWGRPVGRRRRPVRVYP) was synthesized by Biomatik (Kitchener, Ontario, Canada). Briefly, the mixture of 15 μM ThioGlo4 (MilliporeSigma, 59550410MG), 150 mM NaCl, 25 mM HEPES (pH 7.5), and 0.001 % TritonX was reacted with 0.5, 1, 2, 5, and 10 μM of CoA (Sigma, C4780), respectively, for standard curve. Besides, 15 μM ThioGlo4, 50 μM AcCoA (Sigma, A2181), 50 μM Substrate (EEEEIA24), 150 mM NaCl, 25 mM HEPES (pH 7.5), and 0.001% Triton X was reacted with 25, 50, 100, 200 nM of purified NAA10, respectively. All the experiments were performed at 25 °C in duplicate, and the fluorescence intensity was measured with an excitation wavelength of 400 nm, and an emission wavelength of 465 nm using a FlexStation^®^3 multi-mode microplate reader. The fluorescence was continuously recorded for 30 minutes with an interval of 30 seconds. The baseline was subtracted, and initial velocity was calculated using SoftMax Pro Software.

### Adenovirus generation

NAA10-P2A-HaloTag was cloned based on pH6HTC His_6_HaloTag^®^ T7 Vector (Promega, Madison, WI, USA). The NAA10-P2A-HaloTag sequence was inserted into pENTR/D-TOPO vector (Invitrogen) and thereafter into pAd/CMV/V5/DEST vector (Invitrogen) following the manufacturer’s protocol. The vector was digested with Pac I, and the Pac I-digested vector was transfected to HEK293A cells with Lipofectamine 3000 on a 6-well plate. Thereafter, the virus was amplified by infecting HEK293A cells on a 10-cm dish. The crude stock was used in experiments and is designated as Ad-NAA10.

### Ca^2+^ imaging

Single iPSC-CMs were seeded on PDMS-coated micro-patterned coverslips. After 3 days, the coverslips were incubated with 5 μM Fura-2 (ThermoFisher, F14185) at 37°C with 5% CO2 for 20 minutes, and, after washing, placed in a C-Stim CMC microscope chamber (IonOptix) and a temperature of 36–37°C was maintained by using a mTCII Temperature Controller (IonOptix) to circulate extracellular by a closed-loop controller. Extracellular buffer containing (in mM) NaCl 140, KCl 5.4, MgCl2 1.2, CaCl2 1.8, HEPES 10, Glucose 10, and sodium pyruvate 2, with pH of 7.4, was used in this experiment. The samples were imaged using IonOptix Calcium Imaging system installed on an Olympus IX71. During the imaging, the cells were stimulated by MyoPacer (IonOptix). Cells were paced at both 0.5Hz and 1Hz to determine the relative change impairment in calcium reuptake. The end-diastolic levels during each pacing frequency was compared to the baseline prior to pacing. The background was subtracted, and the data were analyzed using IonWizard software (IonOptix).

### Cell-surface Biotinylation

After washing with PBS (+) (PBS containing 0.2mM CaCl_2_ and 1.5mM MgCl_2_), cells were incubated with 0.5 mg/mL Sulfo-NHS-SS-Biotin (Thermo) in PBS(+) for 1 hour on ice with occasional shaking. Thereafter, the biotinylating reaction was quenched by washing 3 times with PBS(+) containing 100 mM glycine. Cells were lysed with mTOR buffer, and part of the lysate was saved as total protein. The rest of the lysate was incubated with Pierce Streptavidin Magnetic Beads (Thermo) at 4 °C overnight on a rotator. The magnetic beads were collected on a magnetic stand, and the protein sample was eluted by incubating with SDS-PAGE, reducing sample buffer at 96–100 °C for 5 minutes.

### Bioinformatics

A multiple sequence alignment of NAA10 sequence was performed with Clustal Omega^60^, and the annotation of conservation was conducted with Jalview^60,61^. DynaMut2 was used to predict the impact of the mutation on protein dynamics and stability^62^. Information of protein structure was obtained from the PDB database, and the human NatA amino-terminal acetyltransferase complex (PDB code: 6C9M)^63^, was applied to the analysis.

### Statistics

Prism 9 (GraphPad, San Diego, CA, USA) was used for statistical analysis. Normal distribution was tested with the Shapiro-Wilk test. For normally distributed samples, data were presented as mean ± SEM. An unpaired Student’s t-test or one-way analysis of variance (ANOVA), followed by Dunnett’s comparison test, was used for two- or more than two-group comparisons. For repetitive measurements, a two-way repeated measures ANOVA, followed by Dunnett’s comparison test, was performed. For samples without normal distribution, data were presented with violin plots. Mann-Whitney test or Kruskal-Wallis test, followed by Dunn’s multiple comparisons test, was conducted for two- or more than two-group comparisons. Results were considered statistically significant at p < 0.05.

## Data Availability

Electrophysiology and other physiology data is available on the Dryad database with the following link, https://doi.org/10.5061/dryad.280gb5mvz. Gene sequencing and patient-related clinical data cannot be shared openly because of risk to patient privacy. Given the rarity of NAA10-related disorders, specific patient information and sequence data may identify patients and family members, breaching patient confidentiality. These data are available upon request with an appropriate and approved IRB protocol at the requesting institution.
